# DICOM file format has better radiographic image quality than other file formats: an objective study

**DOI:** 10.1590/0103-6440202305499

**Published:** 2023-10-27

**Authors:** Murilo Miranda-Viana, Rocharles Cavalcante Fontenele, Fernanda Nogueira-Reis, Amanda Farias-Gomes, Matheus L Oliveira, Deborah Queiroz Freitas, Francisco Haiter-Neto

**Affiliations:** 1 Department of Oral Diagnosis - Oral Radiology Area, Piracicaba Dental School, University of Campinas, Piracicaba, SP, Brazil.; 2 Oral Radiology Area, Dental School, Federal University of Mato Grosso do Sul, Campo Grande, MS, Brazil.

**Keywords:** dental digital radiography, diagnostic imaging, health technologies

## Abstract

This study aimed to assess the influence of the file format on the image quality parameters (image noise, brightness, and uniformity) of periapical radiographs acquired with different digital systems. Radiographic images of an acrylic phantom were acquired with two digital systems - Digora Toto and Express, and exported into five different file formats - TIFF, BMP, DICOM, PNG, and JPEG. Image noise, image brightness (mean of gray values), and image uniformity (standard deviation of gray values) were evaluated in all images. A two-way analysis of variance with Tukey’s test as a post-hoc test was used to compare the results, considering the file formats and radiographic systems as the studied factors. A significance level of 5% was adopted for all analyses. The DICOM image file format presented lower image noise, higher brightness (higher mean gray values), and greater image uniformity (p<0.001) than the other file formats, which did not differ from each other for both digital radiography systems (p>0.05). The Express system revealed lower image noise and greater image uniformity than the Digora Toto system regardless of the image file format (p<0.001). Moreover, the Express showed higher brightness than the Digora Toto for all image file formats (p<0.001), except for the DICOM image file format, which did not significantly differ between the digital radiography systems tested (p>0.05). The DICOM image file format showed lower image noise, higher brightness, and greater image uniformity than the other file formats (TIFF, BMP, PNG, and JPEG) in both digital radiography systems tested.

## Resumo

Este estudo teve como objetivo avaliar a influência do formato do arquivo nos parâmetros de qualidade de imagem (ruído de imagem, brilho e uniformidade) de radiografias periapicais adquiridas com diferentes sistemas digitais. As imagens radiográficas de um fantoma de acrílico foram adquiridas com dois sistemas digitais - Digora Toto e Express, e exportadas em cinco formatos de arquivo diferentes - TIFF, BMP, DICOM, PNG e JPEG. O ruído da imagem, o brilho da imagem (média dos valores de cinza) e a uniformidade da imagem (desvio padrão dos valores de cinza) foram avaliados em todas as imagens. A análise de variância (ANOVA) bidirecional com o teste post-hoc de Tukey foi aplicada para comparar os resultados, considerando os formatos de arquivo e os sistemas radiográficos como os fatores estudados. Um nível de significância de 5% foi adotado para todas as análises. O formato de arquivo de imagem DICOM apresentou menor ruído de imagem, maior brilho (maiores valores médios de cinza) e maior uniformidade de imagem (p<0,001) do que os outros formatos de arquivo, que não diferiram entre si para ambos os sistemas de radiografia digital (p>0,05). O sistema Express apresentou menor ruído de imagem e maior uniformidade de imagem que o sistema Digora Toto, independentemente do formato do arquivo de imagem (p<0,001). Além disso, o Express mostrou maior brilho do que o Digora Toto para todos os formatos de arquivo de imagem (p<0,001), exceto para o formato de arquivo de imagem DICOM, que não diferiu significativamente entre os sistemas de radiografia digital testados (p>0,05). O formato de arquivo de imagem DICOM mostrou menor ruído de imagem, maior brilho e maior uniformidade de imagem do que os outros formatos de arquivo (TIFF, BMP, PNG e JPEG) em ambos os sistemas de radiografia digital testados.

## Introduction

The DICOM image file format is widely recognized as the standard format for the transmission of radiological images and medical information and has a special relevance for computed tomography to standardize different file formats from multiple machine brands and to allow the use of a single DICOM viewing software ^1^. Since the introduction of digital systems, image radiographs have been transmitted online between professionals or between professionals and patients ^2^. However, the different sizes of these file formats must be taken into consideration because of the need for a secure and spacious digital storage media due to the large number and file sizes of the acquired images. 

Image file formats have different levels of data compression and, consequently, the file size in bytes may vary. Several previous studies have investigated the influence of the compression level of digital periapical radiographic images on the subjective evaluation of various diagnostic tasks, such as root resorptions, caries lesions, root fractures, and periapical lesions ^3^ ^,^ ^4^ ^,^ ^5^ ^,^ ^6^ ^,^ ^7^ ^,^ ^8^. Although most studies ^3^ ^,^ ^4^ ^,^ ^5^ ^,^ ^15^ have found no influence between the degree of compression of the images and diagnostic accuracy of the aforementioned clinical conditions, there is still a gap in the literature regarding a consistent explanation for these results. One of the gaps is not including the DICOM image file format in the evaluations, even being recommended as the file format for medical imaging transmission ^1^. Also, to the best of our knowledge, no studies have proposed to perform an objective analysis to evaluate the quality of radiographic images with different file formats, which can provide a better understanding regarding the relationship between radiographic image quality (noise, brightness, and uniformity) and their several file formats, besides being able to justify the non-influence of this factor on the diagnostic accuracy of several clinical tasks, as previously mentioned. Thus, it would be possible to recommend to clinicians the best file format for interpretation and transmission among professionals.

With the progress of understanding about performing objective analysis to investigate the quality of radiographic and tomographic images, it has been possible to investigate the influence of various factors on the images’ density, contrast, and noise level ^9^ ^,^ ^10^ ^,^ ^11^ ^,^ ^12^ ^,^ ^13^ ^,^ ^14^. The objective analysis allows calculating the mean and standard deviation of gray values of the radiographic images ^10^ ^,^ ^14^. From this method, it is possible to measure image noise (mean of the standard deviation (SD) of gray values), image brightness (mean of gray values), and image uniformity (SD of gray values) ^10^ ^,^ ^11^ ^,^ ^14^. Therefore, the current study aimed to objectively assess the influence of the file format on the image quality parameters (image noise, brightness, and uniformity) of periapical radiographs acquired with different digital systems. The null hypothesis stated was that the file formats do not influence the image quality parameters (image noise, brightness, and uniformity) regardless of the digital system tested.

## Material and methods

### Image acquisition and export

For the objective assessment of image quality, an acrylic block measuring 3.0 cm height × 4.0 cm length × 2.0 cm width was used. As a homogeneous material with relatively low X-ray attenuation, the purpose of this block was to simulate the attenuation and scattering of the X-ray beam from soft tissues ^9^ ^,^ ^10^ ^,^ ^12^ ^,^ ^14^. Radiographic images of this phantom were acquired using two radiographic systems with distinct technologies:

1. Phosphor plate (PSP): Express system (Instrumentarium Imaging, Tuusula, Finland), size 2, 8-bit contrast resolution, theoretical spatial resolution of 17.0 lp mm−1.
2. CMOS sensor: Digora Toto system (Soredex, Tuusula, Finland), size 2, 12-bit contrast resolution, and theoretical spatial resolution of 26.3 lp mm−1.

**Figure 1:**
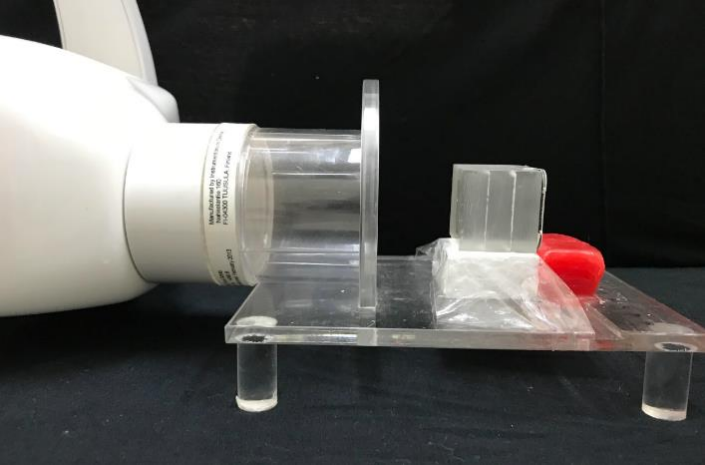
Representation of the standardization of radiographic acquisitions with the acrylic phantom and the image receptor.

**Figure 2:**
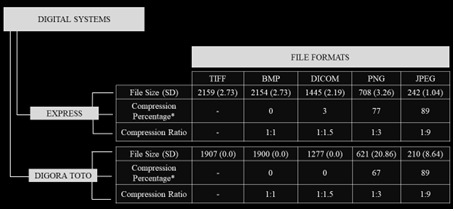
Flowchart of mean (standard deviation) values of the file sizes (in kilobytes), compression percentage, and compression ratio from the images evaluated within each digital radiography system and file format tested. *Compared with TIFF file format; SD, Standard deviation.

**Figure 3:**
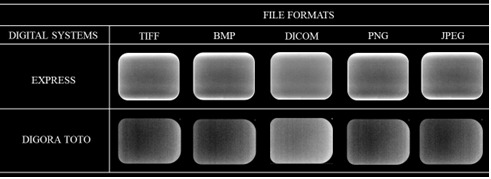
Radiographic images were acquired in the different digital radiographic systems with each image file format investigated.

All images were obtained using the same X-ray unit (FocusTM - Instrumentarium Dental Inc., Milwaukee, WI, USA), under the same acquisition protocol: 60 kV, 7 mA, and exposure time as recommended by the receptors' manufacturers (0.3 s for Express, and 0.18 s for Digora Toto). The default image settings, in both radiographic systems, were established with no auto-filters applied upon acquisition of the periapical images. An acrylic apparatus containing a fixed locator ring was used to standardize the position of the phantom and image receptor according to the paralleling technique (focus-receptor distance of 30 cm, object-receiver of 1.5 cm, as well as vertical angulation of 0° and horizontal of 90°) (Fig. 1). Six repeated radiographic images were obtained with each digital system for reproducibility purposes. Then, each image was exported from the native software: PSP Express - CliniView (Instrumentarium Imaging, Tuusula, Finland) and CMOS - Scanora (Soredex, Tuusula, Finland) into five distinct file formats - TIFF, BMP, DICOM, PNG, and JPEG - totaling 60 images (2 radiographic systems × 6 repetitions × 5 file formats). The software automatically established the bit depth at the moment of exporting the radiographic images in both digital systems tested. Figure 2 shows the average file size (in kilobytes) within each digital radiography system and file format tested. In addition, Figure 3 shows a set of the radiographic images acquired in the different digital radiographic systems and exported in the different image file formats investigated.

### Image Assessment

An oral and maxillofacial radiologist with expertise in the objective analysis of radiographic image quality assessed the radiographs in a medical display (MDRC-2124, Barco N.V., Courtray, Belgium) with 1920 × 1200 pixel resolution. Images were individually evaluated in the ImageJ software (National Institutes of Health, Bethesda, Maryland, USA). The objective assessment consisted of measuring the image noise, image brightness, and image uniformity, as follows.

**Figure 4:**
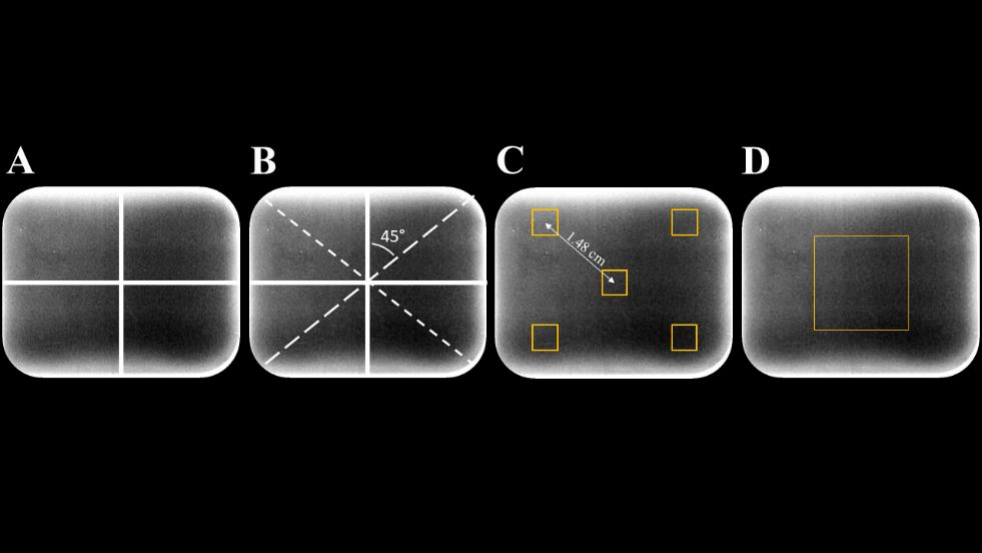
Objective assessment of image quality. (A) - Two lines were determined: one equally dividing the radiographic image into superior and inferior; and a line perpendicular to the first one, equally dividing the image into left and right sides. (B) - Two bisecting lines (45°) were drawn in the crossing area of the first two lines. (C) - Five square ROIs of the same size (4 x 4 mm) were drawn in distinct areas of the image (one ROI in the center of the image, and four ROIs on the bisector lines) to measure image noise. The distance between the center of the central ROI and the center of the other ROIs was standardized in 1.48 cm. (D) - A larger square ROI (1.48 x 1.48 cm) covering the central area of the image was determined to measure the was determined to measure the mean (image brightness) and the standard deviation (image uniformity) of gray values.

Initially, two lines were established on the image: a horizontal line dividing the image equally in superior and inferior aspects, and another line, perpendicular to the first one, dividing the image equally on the left and right sides (Fig. 4A). Then, two bisecting lines (45º) were established on the crossing of the previously described lines (Fig. 4B). To assess different areas of the image, five square regions of the interest (ROIs) measuring 4 × 4 mm were determined: one ROI centered in the area of intersection between the first two lines described, and another four ROIs determined symmetrically on the bisector lines (i.e., upper, and lower corners, in both right and left sides of the image) (Fig. 4C). The distance between the center of the central ROI and the center of the other four ROIs was standardized in 1.48 cm (Fig. 4C). To measure image noise, the SD of gray values of five ROIs was averaged. A more homogeneous image (i.e. with a lower standard deviation) is an image with less noise. Thus, by evaluating the SD of several regions of the radiograph (5 regions distributed over the radiograph's surface), it is possible to determine if an image has higher or lower noise. Consequently, higher values of the SD of gray values of these ROIs reveal higher image noise. Subsequently, a single and larger square ROI (1.48 cm × 1.48 cm) covering the central area of the image was established (Fig. 4D). The mean and the SD of gray values of this ROI were measured to assess the image brightness and image uniformity, respectively. Higher values of mean and SD of gray values reveal higher image brightness (less dark) and lower uniformity, respectively. The analyses were performed in 8-bit images.

### Statistical Analysis

Data were analyzed in the Statistical Package for Social Sciences software v. 24.0 (IBM Corp., Armonk, NY). The results were summarized as mean and SD and compared by two-way analysis of variance with Tukey’s test as a post-hoc test, considering the file formats and radiographic systems as the studied factors. A significance level of 5% was adopted for all analyses. The power of analysis was 95%. 

## Results

The results obtained from the objective analysis are shown in Table 1. There was a statistically significant influence of the different image file formats and the different digital radiography systems investigated on all the image quality parameters evaluated (p<0.001).

**Table 1:** Mean (standard deviation) of image noise, image brightness, and image uniformity according to the digital system and file format.

File format	Image noise (Mean SD gray values of 5 ROIs)		Image brightness (Mean gray values of Central ROI)		Image uniformity (SD gray values of Central ROI)	
*Toto*	*Express*	*Toto*	*Express*	*Toto*	*Express*
	Mean (SD)	Mean (SD)	Mean (SD)	Mean (SD)	Mean (SD)	Mean (SD)
TIFF	21.70 (0.26) Aa	14.10 (0.19) Ba	56.23 (1.80) Bb	93.84 (7.24) Ab	23.60 (0.78) Aa	13.75 (0.28) Ba
BMP	21.91 (0.26) Aa	14.10 (0.19) Ba	56.23 (1.80) Bb	93.84 (7.24) Ab	23.60 (0.61) Aa	13.75 (0.28) Ba
DICOM	20.54 (0.23) Ab	9.89 (0.14) Bb	110.84 (1.26) Aa	117.26 (2.94) Aa	22.35 (0.55) Ab	9.64 (0.19) Bb
PNG	21.91 (0.26) Aa	14.10 (0.19) Ba	56.23 (1.79) Bb	93.84 (7.24) Ab	23.64 (0.62) Aa	13.75 (0.28) Ba
JPEG	22.24 (0.26) Aa	14.28 (0.18) Ba	56.26 (1.79) Bb	93.83 (7.24) Ab	23.85 (0.61) Aa	13.94 (0.27) Ba

SD, standard deviation; ROI, region of interest.

Different uppercase letter show significant difference between digital systems (p<0.001)

Different lowercase letters show significant difference between file formats (p<0.00)

The DICOM image file format showed significantly lower image noise (i.e., lower mean SD gray values of the 5 ROIs evaluated) and higher brightness (i.e., higher mean gray values of the central ROI) than the other file formats, which did not differ significantly from each other irrespective of the digital radiography system (p>0.05). Furthermore, the DICOM image file format showed greater image uniformity (i.e., lower SD of central ROI) than the other file formats, which did not differ significantly from each other for both digital radiography systems (p<0.001). 

Concerning the different digital radiography systems, the Express showed lower image noise and greater image uniformity than Digora Toto regardless of the image file format (p<0.001). Moreover, the Express showed higher brightness than Digora Toto for all image file formats (p<0.001), except for the DICOM image file format, which did not show a statistically significant difference between the digital radiography systems tested (p>0.05). 

## Discussion

The increasing need for online image transmission, aimed at optimizing clinical working time and reducing possible cross-contamination, requires a clear understanding of how the inherent levels of data compression caused by different image file formats can influence image quality parameters in digital radiographic systems. This understanding is crucial for professionals seeking to save storage space without compromising image quality. Moreover, while DICOM is the recommended format for transmitting medical images, a potential difference in image quality between this file format and other available image formats (such as TIFF, BMP, PNG, and JPEG) was unknown. Thus, an objective analysis would provide evidence regarding the null hypothesis that different image file formats do not influence quality. This analysis would complement previous studies that relied on subjective analyses while investigating various diagnostic tasks. Nevertheless, the objective analysis conducted in the present study rejected this hypothesis by demonstrating that the DICOM image file format exhibited significantly lower image noise, higher brightness, and greater image uniformity compared to other formats, which did not differ regardless of the digital radiography system.

These findings regarding DICOM suggest that it offers superior image quality in terms of the evaluated parameters. In contrast, previous subjective analyses of various diagnostic tasks, such as radiographic diagnosis of proximal carious lesions, external and internal root resorption, and vertical root fracture ^3^ ^,^ ^4^ ^,^ ^5^ ^,^ ^8^ ^,^ ^15^ have not identified significant differences in technical factors that affect digital image quality parameters among different formats. However, it is important to note that these evaluations did not include the DICOM format, highlighting the need to incorporate this image file format in future studies focusing on different diagnostic tasks.

Concerning the two digital radiographic systems tested, the Express system presented lower image noise, higher brightness, and greater image uniformity, resulting in images with a more uniform pixel value and less density than those from the Digora Toto. This finding aligns with a previous study that conducted an objective analysis of radiographs obtained after adding a lead foil to these two digital radiographic systems ^9^. Similarly, it was hypothesized that Digora Toto would produce a more significant amount of secondary radiation due to its higher number of electronic components and greater physical thickness than the Express system. Another factor discussed earlier suggests that the lower signal-to-noise ratio may also explain the higher radiographic sensitivity and noise for the Digora Toto system. Due to the difference in exposure time recommended by each manufacturer, Digora Toto used a shorter time for image acquisition, which should have a lower signal-to-noise ratio than the Express system and could interfere with image quality. A previous objective analysis proved that longer exposure times produce images of significantly lower brightness using intraoral digital radiographic systems, including Express and Digora Toto systems ^16^. Also, the different spatial resolutions may be another factor to justify the statistically significant difference observed between the tested digital radiographic systems. For instance, the Digora Toto system produced images of 668 dpi regardless of the file format. Conversely, the Express system produced images of 726 dpi. Thus, the higher spatial resolution observed for the Express system could justify the better image quality parameters noticed in the current investigation for this system, as revealed by the lower noise, higher brightness, and greater image uniformity. It is essential to highlight that the authors chose to adhere to the manufacturer’s exposure time recommendations. This decision was made considering the different sensitivities of the receptors and aiming at maintaining a scenario as close as possible to clinical practice.

Another interesting result is that the Digora Toto system presented lower brightness due to the lower values of the mean gray values achieved by the assessment of a central broad area of the radiographic images for all image file formats, except for the DICOM image file format. An important aspect to be highlighted, given the positive results presented by the DICOM image file format, is related to its size. Its intermediate size, on average (1.445 kB for Express and 1.277 kB for Digora Toto), smaller than the TIFF (2.985 kB for Express and 1.907 kB for Digora Toto) and BMP (2.890 kB for Express and 1.900 kB for Digora Toto) file formats, reinforce its great cost-benefit statement for online transmission and digital storage in the clinical practice. Even with a compression ratio of 1:1.5, the DICOM file format showed better image quality parameters than other file formats with lower compression rates. It is hypothesized that the DICOM format is not susceptible to the post-processing factors concerning the image visualization, such as the display device resolution and the native setting of the image viewer software ^1^. Thus, the recommendation of applying the DICOM file format to visualize and interpret radiographic images can be considered worthwhile based on the results achieved by the current investigation. In addition, the lower image noise, greater uniformity, and no difference between the digital radiographic systems in brightness, the DICOM format seems to have improved the radiographic brightness regardless of the system.

Due to technological advances, online transmission and interpretation of radiographic images using portable devices (e.g., smartphones and tablets) are becoming increasingly common in clinical routine ^2^ ^,^ ^17^ ^,^ ^18^. Although our results indicate that the DICOM format objectively presents better image quality than the other file formats tested, its use on handheld devices is still challenging. The DICOM file is not recognized by the graphic system of these devices for immediate visualization of the image. Thus, it is necessary to load the DICOM image into secondary software to allow adequate visualization, which hinders the practical process of radiographic analysis in these devices ^19^. Conversely, direct visualization of images in JPEG file format is possible using handheld devices due to its recognition by the graphics systems of the handheld devices, smaller file size, and fast transmission ^4^ ^,^ ^15^. A previous investigation ^4^ proved that online streaming of radiographic images in JPEG format did not impair caries lesion diagnosis. However, the DICOM format was not included in this investigation. Thus, it is encouraged that future studies investigate whether the DICOM file subjectively improves the evaluation of different diagnostic tasks compared to other widely used formats (TIFF, BMP, PNG, and JPEG).

To summarize, the DICOM image file format showed lower noise, greater uniformity, and higher brightness than the other file formats tested in both digital radiography systems, and the digital systems influenced the objective image quality analyses for all formats. This last result followed previous studies in the scientific literature ^10^ ^,^ ^11^ ^,^ ^14^, although other intraoral radiographic systems should be tested to evaluate the current results' reproducibility. Even though the present study has the limiting factor of being an *in-vitro* study, the result that there is a difference in image quality in different file formats for the two systems evaluated can be used as a reference for future diagnostic studies to verify the real impact of this finding in a clinical setting. Furthermore, this clarification will assist oral radiologists and clinicians in prioritizing the optimal image quality format and digital system for transmitting digital examinations. In conclusion, the DICOM image file format showed lower image noise, higher brightness, and greater image uniformity than the other file formats (TIFF, BMP, PNG, and JPEG) in both digital radiography systems tested.
